# Probing the role of thermal vibrational disorder in the SPT of VO$_2$ by Raman spectroscopy

**DOI:** 10.1038/s41598-020-79758-1

**Published:** 2021-01-15

**Authors:** Aminat Oyiza Suleiman, Sabeur Mansouri, Nicolas Émond, Boris Le Drogoff, Théophile Bégin, Joëlle Margot, Mohamed Chaker

**Affiliations:** 1 Institut National de la Recherche Scientifique, Énergie Matériaux Télécommunications, 1650, Boulevard Lionel-Boulet, Varennes, QC J3X 1S2 Canada; 2 Department of Materials Science and Engineering, Massachusetts Institute of Technology, 77 Massachusetts Avenue, Cambridge, MA 02139 USA; 3 Département de Physique, Complexe des Sciences, Université de Montréal, 1375 Avenue Thérèse-Lavoie-Roux, Montréal, QC H2V 0B3 Canada

**Keywords:** Engineering, Materials science, Physics

## Abstract

Phase competition in transition metal oxides has attracted remarkable interest for fundamental aspects and technological applications. Here, we report a concurrent study of the phase transitions in undoped and Cr-doped VO$_2$ thin films. The structural, morphological and electrical properties of our films are examined and the microstructural effect on the metal–insulator transition (MIT) are highlighted. We further present a distinctive approach for analyzing the Raman data of undoped and Cr-doped VO$_2$ thin films as a function of temperature, which are quantitatively correlated to the electrical measurements of VO$_2$ films to give an insight into the coupling between the structural phase transition (SPT) and the MIT. These data are also combined with reported EXAFS measurements and a connection between the Raman intensities and the mean Debye–Waller factors $\sigma ^2$ is established. We found that the temperature dependence of the $\sigma _{R}^{2}(V-V)$ as calculated from the Raman intensity retraces the temperature profile of the $\sigma _{EXAFS}^{2}(V-V)$ as obtained from the EXAFS data analysis. Our findings provide an evidence on the critical role of the thermal vibrational disorder in the VO$_2$ phase transitions. Our study demonstrates that correlating Raman data with EXAFS analysis, the lattice and electronic structural dynamics can be probed.

## Introduction

Vanadium dioxide (VO$_2$) is a typical correlated electron material, which exhibits a reversible first-order metal–insulator transition (MIT) at a relatively low temperature $\sim$ $68 \,^{\circ }\hbox {C}$. Upon heating, the VO$_2$ switches from an insulating monoclinic phase (M1 or M2) to a metallic tetragonal rutile (R) phase^1–4^. This MIT, with a huge change in the conductivity of up to 5 orders of magnitude, has been attracting considerable interest for fundamental aspects^5–8^, and for potential applications^9–18^. This unique property positions VO$_2$ at the forefront of exploitable new technologies such as in micro/nanoelectronic and photonic applications that include thermal control systems^9,10^, microbolometers^11^, optical limiters^12^, ultra-fast optical switches^13^, gas sensors^14^, nanoactuators^15^, smart windows^16^, thermochromic devices^17^, and MIT transistors^18,19^. However, to efficiently implement VO$_2$ in functional and optimized devices, the microscopic origin of the MIT needs to be elucidated, as it is still debated. Is it a Peierls-like structural phase transition (SPT) mechanism where the MIT is driven by instabilities in electron-lattice dynamics or a Mott transition where strong electron-electron correlations drive charge localization and collapse of the lattice symmetry^5–8^? Recent progress in VO$_2$ MIT mechanism points to a key role of lattice vibrations and attribute the metallization of vanadium dioxide to a large phonon entropy^20^. In addition, using extended x-ray absorption fine structure (EXAFS), Hwang et al.^21^ revealed a significant increase in the Debye–Waller factors of the vanadium–oxygen (V–O) and vanadium–vanadium (V–V) pairs in the (111) direction at the MIT. This thermal disorder is attributed to the phonons of the V–V pairs in the same direction. There is, thus, some real interest in the understanding of the role of the thermal vibrational disorder in the phase transition and how this factor could influence the M1-R and the M2-R phase transitions. This can be addressed by studying the electrical and lattice-dynamic properties of VO$_2$, which offers useful information in the understanding of the MIT in VO$_2$, since combining electrical and Raman data (more sensitive to the structural transition) we can get concurrently information about the electronic and the structural transitions.

Raman spectroscopy probes the local structural properties of materials based on the characteristic of their vibrational modes (frequencies, widths and intensities) and it has already been successfully used to study the coupling between charge and lattice degrees of freedom in strongly correlated electron systems^22,23^ and observation of low-fequency spin excitation^24^. Moreover, the evolution of phonon intensities as a function of temperature provides crucial information on the electronic properties as well as the bonding covalence and the local thermal disorder. The Raman data can be combined with EXAFS measurements for a deeper understanding of the local lattice properties of strongly correlated electron materials. The approach is based on the analysis that the bond-stretching vibration of the atoms, which also contributes to the reduced Raman intensity, is fundamentally the vibrational mechanism determining the EXAFS Debye–Waller factors^25^. Parameterizing EXAFS measurements in terms of structural quantities provide information on the average near-neighbor distances, its mean-square relative displacements (MSRD) $\sigma^2$, and the coordination numbers N^25^. More specifically, the $\sigma^2$ of the central absorbing atom relative to its neighbors, which appears in the Debye–Waller (DW) factor $\exp$(− 2k$^2$ $\sigma^2$), is crucial in the EXAFS analysis. Its temperature dependence determine the thermal contribution to DW factor and inform on the local structural and thermal vibrational disorder.

In this work, we have stabilized the M2 intermediate monolinic phase by doping VO$_2$ with chromium (Cr) using reported methods^26,27^. Thus, by studying concurrently the M1-R and the M2-R phase transitions, it is expected that a valuable information will be provided to better understand the VO$_2$ MIT driving force. We present a distinctive approach for analysing Raman data of the undoped (M1) and Cr-doped (M2) VO$_2$ thin films as a function of temperature. Films with different thicknesses are deposited on c- and r-plane sapphire substrates. The crystallinity and morphology of the films are examined by XRD and AFM techniques. The Raman measurements are correlated to the four-probe resistivity measurements to learn more about the coupling between the SPT and the MIT, which is accomplished by deconvoluting the Raman spectra of VO$_2$ films to extract the $r/(m+r)$ ratio where *m* and *r* are the fitting parameters relative to the monoclinic and the rutile phases, respectively. We also combine Raman data with EXAFS to reveal a connection between the Raman intensities and the mean Debye–Waller factors, $\sigma^2$. We find that the temperature dependence of the $\sigma_R^2$ (V–V) as obtained from the reduced Raman intensity reproduces the temperature profile of the $\sigma_{EXAFS}^2$ (V–V) measurements as deduced from the EXAFS data therefore providing a clear evidence of the role of the thermal vibrational disorder in the SPT.

## Results and discussion

**Figure 1:**
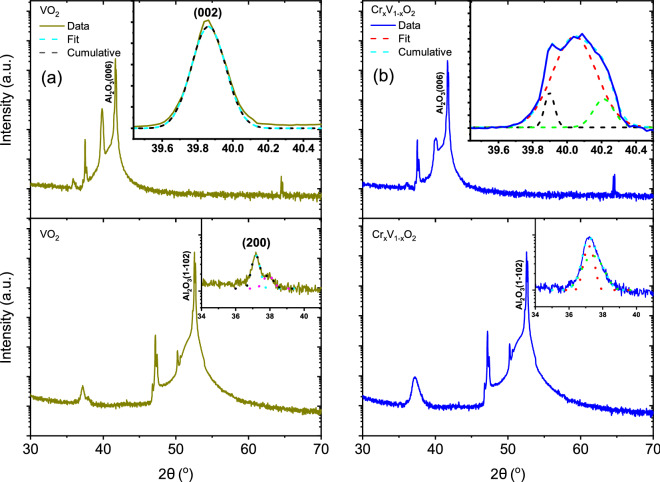
$\theta - 2\theta$ scans of undoped (**a**) and Cr-doped VO$_2$ (**b**) films deposited on c- and r-plane sapphire substrates with their respective insets showing the main peak positions.

Figure 1 shows the $\theta - 2\theta$ XRD patterns from 140 nm undoped and Cr-doped VO$_2$ samples deposited on c- and r- plane sapphire substrates, along with the fitting of the VO$_2$ peak with a Gaussian function in the inset. The stoichiometry of Cr$_x$V$_{1-x}$O$_2$ ($x = 0.03$) is determined from Rutherford backscattering spectroscopy. The main 2$\theta$ positions of our films for the preferential growth along the (002)^28–31^/(020)^32–34^ for the c-sapphire and (200)^35,36^ for r-sapphire were respectively determined to be $39.86^{\circ }$ and $37.17^{\circ }$ for VO$_2$ and $40.04^{\circ }$ and $37.18^{\circ }$ for Cr-doped VO$_2$. The epitaxial growth of the VO$_2$(M1) films along the (200) is confirmed by the good agreement of the peak positions with previous studies^31,34,36^ and the phi-scan (not shown) measurements performed for the VO$_2$ (210) and (220) off-axis peak indicate the high crystalline quality of the VO$_2$(M1) films on both substrates. No diffraction patterns for heteroepitaxially grown Cr-doped VO$_2$ with M2 phase films have been reported in literature. The evolution of the (002) and (200) peak positions as a function of thickness has been investigated down to 5 nm (see Fig. S1-1 in the Supplementary Material). Deconvolution of the peaks of Cr-doped VO$_2$ on c-sapphire was performed using three Gaussian functions with centers at $39.90^{\circ }$, $40.04^{\circ }$ and $40.21^{\circ }$ while that of VO$_2$ on r-sapphire was done using two Gaussian functions with centers at $37.18^{\circ }$ and $37.41^{\circ }$.

A clear peak shift and broadening was observed for thicknesses from 5 to 140 nm for all samples (see Fig. S1-1 in the Supplementary Material). For example, for undoped VO$_2$ on c-sapphire, the broadening reaches a maximum at a thickness of 30 nm accompanied by a slight shift to lower angles at 140 nm. On the other hand, for doped samples, the broadening also reached a maximum at a thickness of 30 nm ($39.89^{\circ }$) but without any obvious peak shift. In contrast, for Cr-doped VO$_2$ on r-sapphire, the peak sharpens with thickness while shifting to lower angles, from $37.44^{\circ }$ at 5 nm to $37.18^{\circ }$ at 140 nm. Undoped VO$_2$ on r-sapphire showed a broad peak at 5 nm at an angle of $37.96^{\circ }$, which shifts to $37.01^{\circ }$ at 15 nm and remains close to this position for larger thicknesses, just slightly shifting to higher angles. The broadening and shifting of peaks with varying thicknesses is most probably related to the strain effect (tensile or compressive) exerted on the film by the substrate due to lattice mismatch, the lattice parameter of the films getting closer to that of the bulk with increasing thickness.

**Figure 2:**
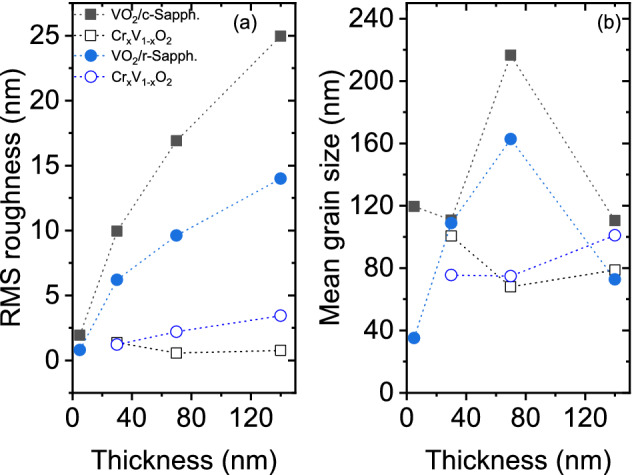
RMS roughness (**a**) and mean grain size (**b**) for various film thicknesses of undoped and Cr-doped VO$_2$ on c- and r-plane sapphire substrates.

Figure 2a,b show the RMS roughness and the mean grain size as determined from the analysis of the AFM measurements performed on VO$_2$ and Cr-doped VO$_2$ films deposited on c- and r-plane sapphire substrates. A slow increase in RMS roughness with thickness is observed for undoped and Cr-doped VO$_2$ on c- and r-plane sapphire substrates.

**Figure 3:**
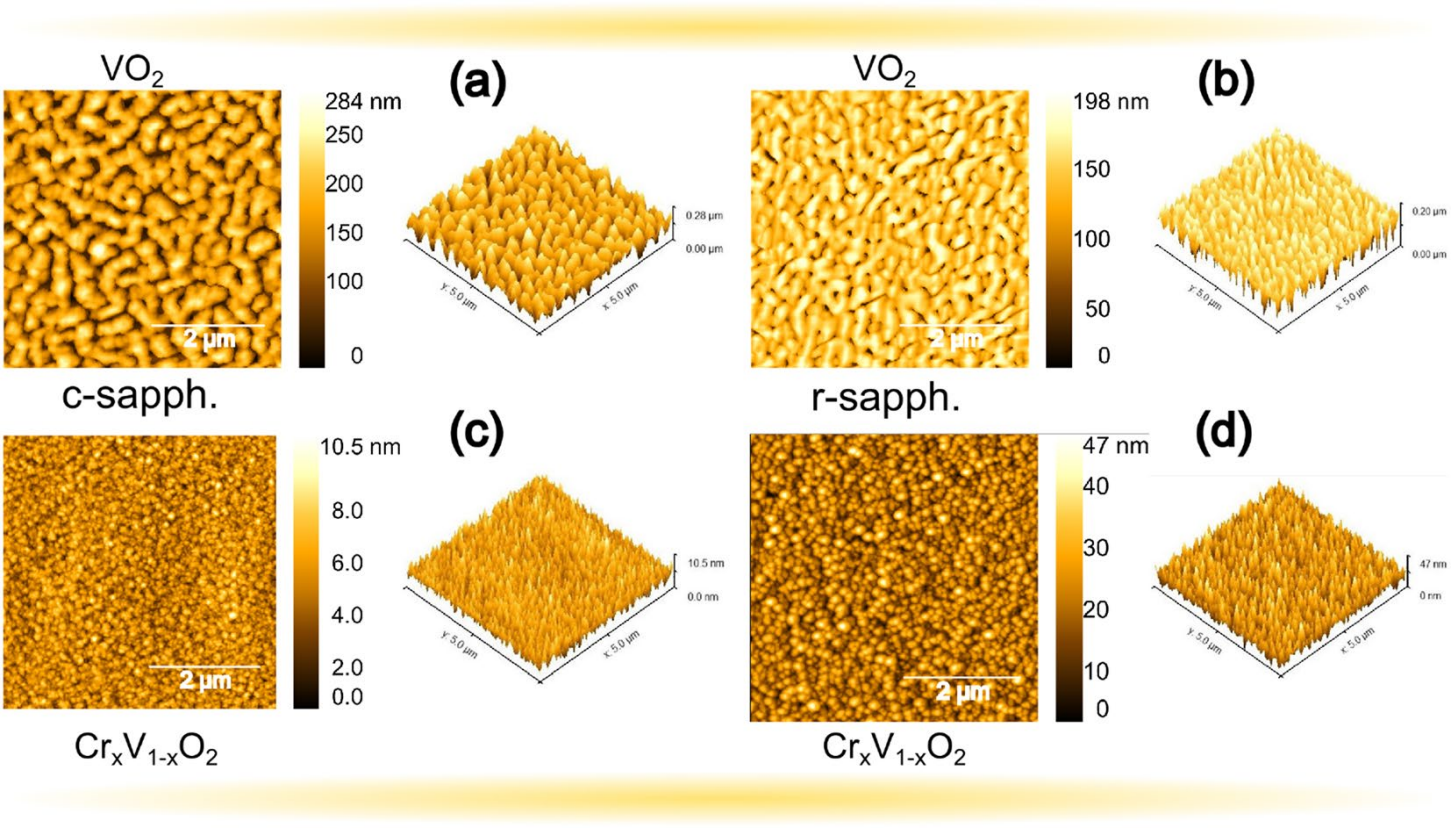
AFM surface morphology of 140 nm-thick undoped (**a**,**b**) and Cr-doped (**c**,**d**) VO$_2$ films on c- and r-plane sapphire substrates.

The temperature-dependent resistivity of single phase undoped (M1 phase) and Cr-doped (M2 phase) 140 nm-thick

**Figure 4:**
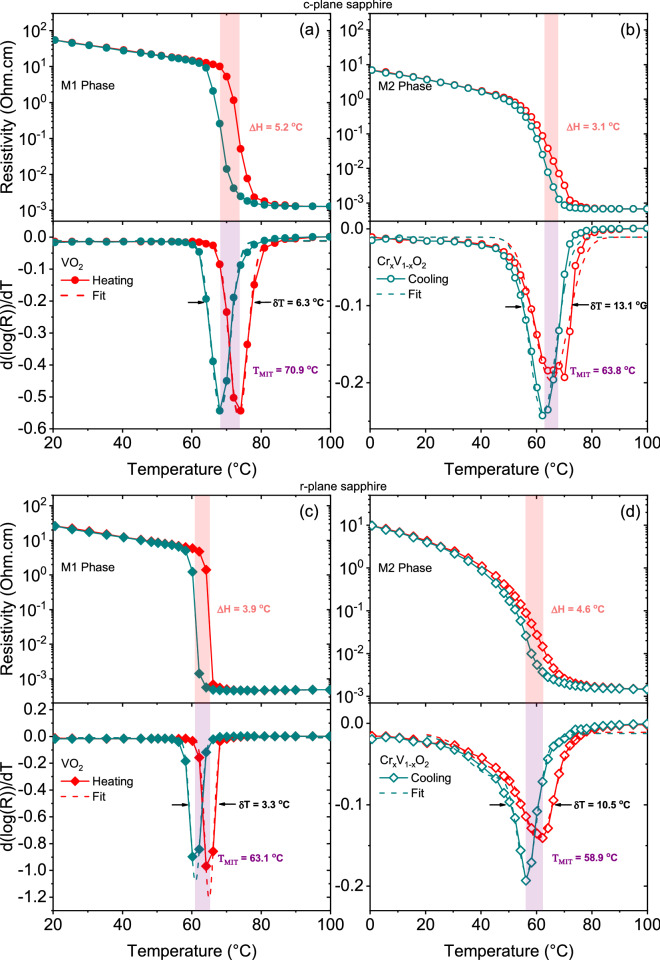
Resistivity and the derivative plot of the temperature-dependent resistivity of undoped (**a**,**c**) and Cr-doped VO$_2$ (**b**,**d**) deposited on c- and r-sapphire for the cooling and heating cycles showing the hysteresis width, $\Delta$H (difference between $\hbox {T}_{\mathrm{MIT}}$ of both thermal cycle), the transition width, $\delta$T (the full-width half-maximum) and the transition temperature, $\hbox {T}_{\mathrm{MIT}}$.

VO$_2$ films on c- and r-plane sapphire substrates is shown in Fig. 4. The transition temperature ($\hbox {T}_{\mathrm{MIT}}$) of VO$_2$ films were generally higher than their Cr-doped counterparts by $7.1\,^{\circ }\hbox {C}$ and $4.2\,^{\circ }\hbox {C}$, respectively. The observed VO$_2$ behaviour is in line with previous studies^37,38^ as a large and sharp transition with a narrow hysteresis is observed for VO$_2$ films grown on c- and r-sapphire substrates, as shown in Fig. 4a,c, where up to 4.6 and 4.7 orders of magnitude change in resistivity were respectively obtained. For Cr-doped VO$_2$ films on c- and r-sapphire, a 4.0 and 3.8 orders of magnitude were respectively obtained compared to the undoped films. Generally, a smaller hysteresis and a larger transition width were recorded for the Cr-doped samples on both substrates as thickness decreases (not shown here). It is evident from Fig. 4a,c that higher and lower $\hbox {T}_{\mathrm{MIT}}$ were obtained on c- and r-sapphire substrates as compared with the bulk value. This result is in agreement with previous studies^39,40^, and is attributed to the compressive/tensile strain experienced along the c$_R$ axis of rutile VO$_2$ deposited on c-/r-sapphire, respectively. Interestingly, for Cr-doped samples, lower $\hbox {T}_{\mathrm{MIT}}$ of $63.8\,^{\circ }\hbox {C}$ and $58.9\,^{\circ }\hbox {C}$ are obtained for films deposited on c- and r-plane sapphire respectively. This observation is in contrast to what is reported in literature^26^, where VO$_2$ with Cr ($\ge {2.4}$ at. %) results in a rise in transition temperature. The observed discrepancy between these two results could be related to the location of the dopant in the lattice, interstitial instead of substitutional since the kind of of defects introduced in the crystal metallic phase is not clear^41^. Pan et al.^41^ showed that the total energy of the system with Cr substitution defects was 8.9% smaller than that with Cr interstitial defects, thus, Cr substitution should result in increment of the phase transition since it gives rise to charge transfer from V to Cr. It could also be due to higher density of grain boundaries for Cr-doped samples, which creates a greater number of nucleating defects limiting the electrical conductivity. These grain boundaries are enhanced by the spherical-like grains present on both substrate’s surfaces compared to the undoped ones (see Fig. 3). The better connection between the grains for Cr-doped samples with regards to undoped ones facilitates the formation of a current path between the electrodes. To confirm the enhanced connection between these grains, optical measurements (not shown here) were performed and the transition temperatures of undoped samples were observed to be higher than that of electrical measurements compared to the doped samples.

**Figure 5:**
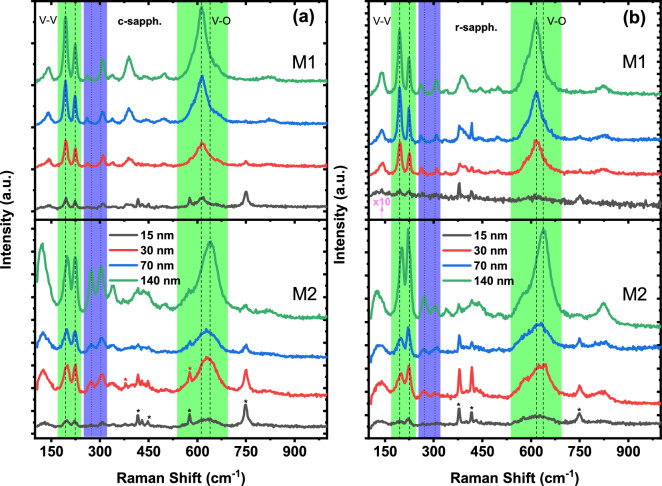
Room temperature Raman spectra of VO$_2$ and Cr-doped VO$_2$ films with different thicknesses on c-plane (**a**) and r-plane (**b**) sapphire substrates. The shaded green areas highlight the V–V and the V–O Raman modes while the shaded blue areas indicate the shift of the M1 Raman modes in the M2 phase in addition to the shift of the V–O Raman modes. The asterisk indicates the respective substrate’s Raman peaks.

Figure 5a,b show the unpolarized Raman spectra of VO$_2$ and Cr-doped VO$_2$ phases as a function of film thickness (140, 70, 30 and 15 nm) deposited on c- and r-plane sapphire substrates. Typical phonon signatures associated with the M1 and M2 monoclinic VO$_2$ phases are observed^27,42,43^. The line widths of phonons are narrow, close to 8 cm$^{-1}$ for the 193 and 224 cm$^{-1}$ modes, confirming the high crystalline quality of our films in agreement with our XRD data. Also, with decreasing thickness down to 15 nm, the typical Raman signature of VO$_2$ was still observed indicating high structural and chemical phase stability of our films. The Raman spectra of VO$_2$ films are numerically calibrated with respect to that of the substrate to avoid any spectrometer readjustment during our measurements. Table 2 lists the phonon frequencies observed for undoped and Cr-doped VO$_2$ on c- and r-plane sapphires for thicknesses down to 15 nm. Based on space group analysis, 18 Raman-active modes are predicted for the M1 phase (9 A$_g$ +9 B$_g$). For the M2 phase, group theory predicts the same number of Raman active modes but with different symmetries (10 A$_g$ + 8 B$_g$)^27^. As expected, similar peak patterns are observed for the M1 and M2 phases but with different peak positions and intensity distributions. In accordance with previous studies, the appearance of the M2 phase is mainly marked by the emergence of the mode at 640 cm$^{-1}$^27,44^. However, in literature, an unequivocal assignment of the phonon symmetries and the ionic displacements is still missing^43,45,46^. Here, our assignment of the phonons is based on a recent study on the assignment of monoclinic VO$_2$ Raman modes^46^. Using isotope substitution and density functional theory calculations, Marini et al.^27^ have demonstrated that the two low-frequency A$_g$ phonons, (193 cm$^{-1}$ and  224 cm$^{-1}$) correspond to V–V lattice motion while all the other vibration modes involve the V–O bonding, especially the mode around 615 cm$^{-1}$ that exhibits the largest shift. These peaks are indicated by the shaded green area in Fig. 5a,b while the shaded blue area highlights the peak shift between the doped and the undoped samples. The high frequency (V–O) Raman modes for 15 and 30 nm Cr-doped VO$_2$ on c-sapphire moved to lower values of 630 cm$^{-1}$ from 646 cm$^{-1}$ and from 637 cm$^{-1}$ to 619 cm$^{-1}$ on r-sapphire for thicker films (70 and 140 nm). A shift from the Raman mode at 613 cm$^{-1}$ (70 and 140 nm) to 615 cm$^{-1}$ (15 and 30 nm) VO$_2$ on c-sapphire was also observed. Similarly, for VO$_2$ on r-sapphire, a shift from 615 to 619 cm$^{-1}$ was observed. The respective redshift and blue shift of peaks for both samples could be attributed to the different strain experienced by the films. Although it is known that a compressive (blue shift) strain is associated with r-plane sapphire while tensile (redshift) strain is associated with c-plane sapphire.

**Figure 6:**
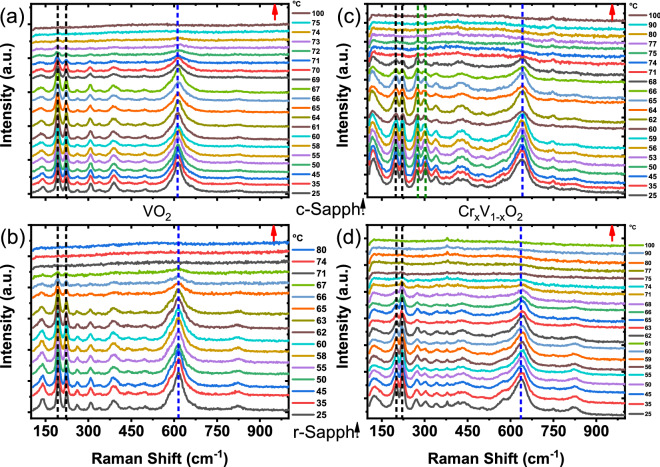
Raman spectra of undoped (**a**,**b**) and Cr-doped (**c**,**d**) VO$_2$ on c-plane and r-plane sapphire substrates with the red arrows indicating the thermal cycle of heating. The dotted black and blue lines depict the Raman peak positions of the low (V–V) and the high (V–O) frequency Raman modes respectively.

Figure 6 shows the Raman spectra of VO$_2$(M1) and the Cr-doped VO$_2$(M2) phases as a function of temperature (heating process) for the films of 140 nm deposited on c- and r-plane sapphire substrates respectively. The Raman spectra obtained for the cooling process are presented in the Supplementary Material (Fig. S1-6). At room temperature, a sharp phonon structure is observed. With increasing temperature up to $60\,^{\circ }\hbox {C}$, the Raman signatures of both VO$_2$(M1) and Cr-doped(M2) phases weaken to finally disappear above $\hbox {T}_{\mathrm{MIT}}$. Similar Raman signatures are recovered during the cooling process when $\hbox {T} < \hbox {T}_{\mathrm{MIT}}$. The disappearance of the peaks at high temperature testifies the structural phase transition to the rutile metallic phase of VO$_{2}$. This huge difference in Raman signatures across the MIT makes Raman spectroscopy a sensitive probe for the structural phase component analysis. Previous quantitative analyses of VO$_{2}$ Raman spectra generally discuss about the temperature dependence of Raman intensity of some specific peaks and rarely consider the whole Raman signature^40,47–49^. These previous studies mainly focus on the modes around 610 cm$^{-1}$ and 200 cm$^{-1}$, since they are still clearly observed across the MIT. Furthermore, in these previous studies the integrated intensity of these Raman lines was determined with respect to the background Raman signal. However, the Raman response within a standard model curve^50^, is not only composed of the phonon contributions, each described by damped harmonic oscillator, but also an electronic contribution which arises from collision-dominated electronic scattering. This makes these previous quantitative analyses not entirely reliable, as metallic domains are already present during the MIT. This is visible in the Raman spectra via an increase in the background signal as observed in our temperature-dependent Raman spectra.

**Figure 7:**
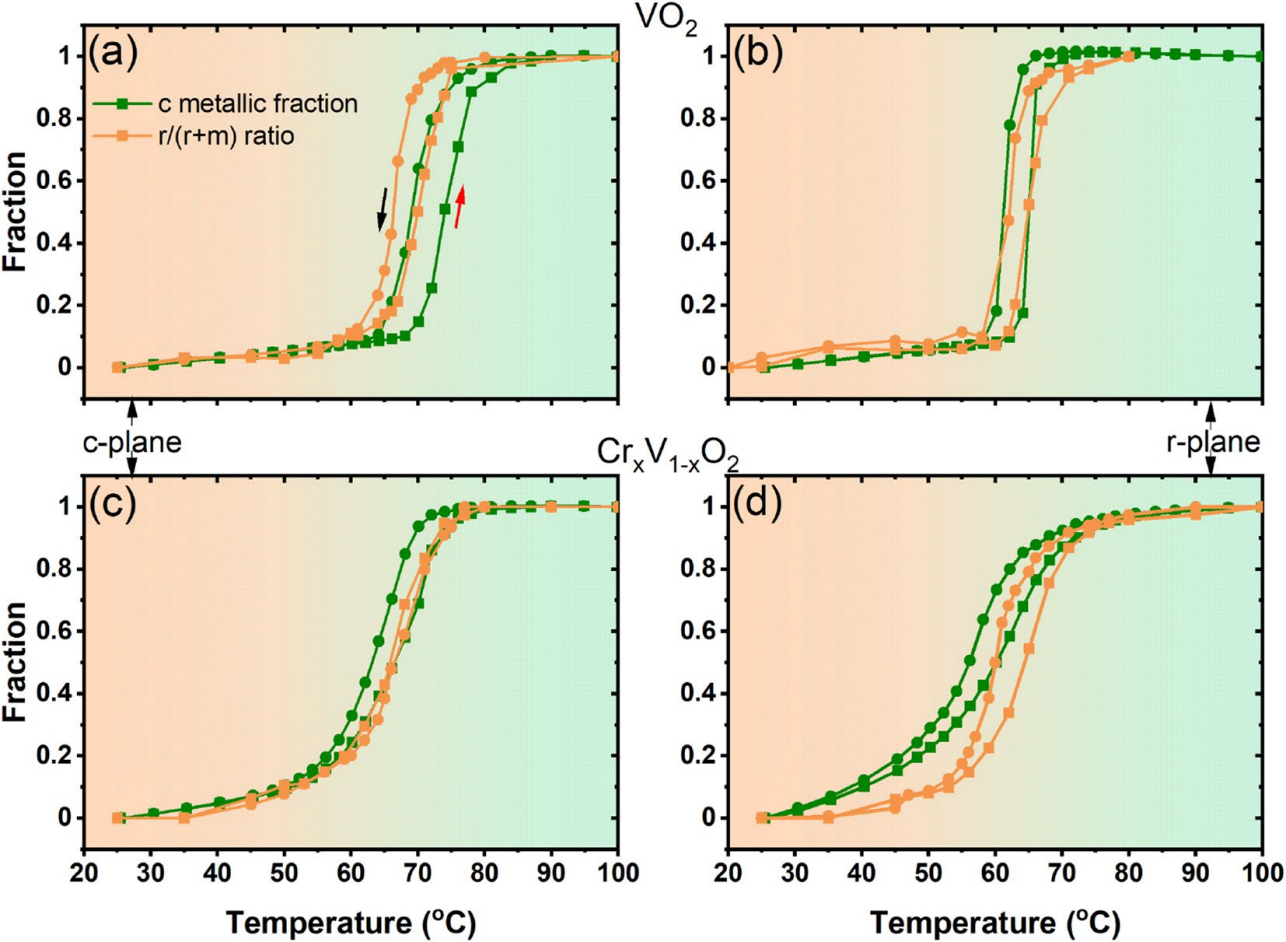
Metallic and rutile fraction analysis of undoped (**a**,**b**) and Cr-doped VO$_2$ (**c**,**d**) on c- and r-sapphire substrates.

Here, we analyze the structural phase changes across the MIT by comparing the phonon signature of mixed phases with that of the pure room and high temperature phases. In this analysis, the Raman spectra at room temperature (RT) and at high temperature (HT), i.e., well above T_MIT_, are considered as the reference for monoclinic and rutile phases respectively. By a Multiple Linear Regression analysis and using Eq. (1), we can find the fitting parameters $m$ and $r$, which correspond to

1 $$\begin{aligned} R_s(T)-B(T)=m[R(RT)-B(RT)]+r[R(HT)-B(HT)], \end{aligned}$$

where $R_s(T)$ and $B(T)$ are the experimental Raman spectrum and its corresponding straight baseline, respectively. Here, the baseline curve is determined from the linear part of the spectrum above 1200 cm$^{-1}$ since the electronic scattering contribution to the Raman response is weak at high frequency. Using the fitting parameters $m$ and $r$, the quantities $m$/($m$+$r$) and $r$/($m$+$r$) correspond to the monoclinic and rutile phase fractions (i.e., the co-existence of highly conductive (rutile) and weakly conductive (monoclinic) phases over a broad T-range across the MIT, even at the nanoscale level as reported by recent studies^51,52^), respectively. Hereafter the calculation of these quantities is called structural fraction analysis.The evolution of rutile phase fraction (the $r$/($m$+$r$) ratio) of VO$_2$(M1) and Cr-doped(M2) phases as a function of temperature for both c- and r-plane sapphire substrates is shown in Fig. 7. The obtained results reproduce similar temperature profile as our electrical measurements and confirm the VO$_2$ reversible MIT between the insulating monoclinic phase and the rutile metallic phase during the heating and the cooling cycles. Here, the transition temperature for the cooling and heating cycles is defined where the $r$/($m$+$r$) ratio equals to 0.5. In order to learn more about the coupling between the SPT and the MIT in both VO$_2$(M1) and the Cr-doped(M2) phases, we compare, in the same figure, the rutile phase fraction for each samples to its corresponding metallic fraction. The metallic fractions are deduced from our electrical measurements using an effective medium approximation^53^ following Eq. (2):

2 $$\begin{aligned} c\dfrac{\log (R_{co} (HT))-\log (R(T))}{\log (R_{co} (HT))+ a \log (R(T))} +(1-c) \dfrac{\log (R_i (RT))-\log (R(T))}{\log (R_i (RT))+a \log (R(T))}=0, \end{aligned}$$

where $c$ and $(1-c)$ are the volume fractions of conductive ($co$) and insulating ($i$) phases respectively while $R(T)$ is the measured resistance. The factor $a$ is related to the depolarizability $q$ by $a = (1-q)/q$. This factor is fixed at $a = 1.5$ as reported in the Refs.^48,54^. In our approach, we take as a variable log$(R)$ since in conventional analysis T_MIT_ is determined using the logarithmic derivative. At each temperature, the volume fraction of the metallic phase $c$ can be calculated using the measured resistance values presented in Fig. 4. Here, the transition temperature for the cooling and heating cycles is also determined when $c$ equals 0.5. This approach makes it possible to find the same transition temperatures coincident with the logarithmic derivative of our resistivity measurements with an adjustment of the equation in Ref.^55^.

Taken together, these results reveal that the coupling between the structural and the electronic transitions in VO$_2$ films is sensitive to the substrate strain (tensile or compressive strain) and to the nature of the involved phase transitions (M1-R or M2-R). The transition temperatures, obtained from metallic and rutile fraction analyses, are summarized in Table 1. A difference in the structural and electronic transition temperature is observed for undoped VO$_2$ on c-plane sapphire substrate as seen in Fig. 7a.

**Table 1:** Transition temperatures determined from fraction analyses of electrical and Raman measurements.

Samples	Process	Metallic fraction analysis ($^{\circ }\hbox {C}$)	Structural fraction analyses ($^{\circ }\hbox {C}$)
VO$_2$ c-plane sapphire	Heating	74	70
	Cooling	69	67
Cr-doped VO$_2$ c-plane sapphire	Heating	67	67
	Cooling	63	67
VO$_2$ r-plane sapphire	Heating	65	65
	Cooling	61	62
Cr-doped VO$_2$ r-plane sapphire	Heating	60	65
	Cooling	56	60

At 0.5, the structural fraction analyses give transition temperatures of $\sim$ 67 and $\sim$ $70\,^{\circ }\hbox {C}$ for cooling and heating cycles respectively, while those from the metallic fraction analysis are 69 and $74\,^{\circ }\hbox {C}$. Also, we find that the electronic transition takes place when the structure is completely rutile. Furthermore, our Raman study in heating (cooling) run, showed an unexpected blue shifts at $\sim$ $70\,^{\circ }\hbox {C}$ ($\sim$ $67\,^{\circ }\hbox {C}$) of the characteristic phonons at 193 cm$^{-1}$ (V–V vibration) and at 614 cm$^{-1}$ (V–O vibration) (results not shown here). The frequency hardening behaviour is similar to that reported in previous studies^40,49^ where it was attributed by the authors to a transient structure, like the M2 phase, stabilized in a narrow temperature range occuring just before the transition to the rutile phase. Okimura et al.^34^ have observed an intermediate insulator phase by in situ temperature-controlled x-ray diffraction across the MIT specifically in VO$_2$ grown on c-plane sapphire substrate, which they assigned to the M2 phase. In agreement with these previous studies, our fraction analysis approach is consistent with the presence of an intermediate insulator structure that may induce a large phase separation around the MIT for the VO$_2$ grown on c-plane sapphire (see Fig. 7a). Actually, the presence of this intermediate phase (a third phase) in the case of VO$_2$ on c-sapphire makes the hypothesis of the co-existence of only two phases invalid and as such the MIT and SPT temperatures cannot be directly compared. We speculate that this intermediate insulator structure, observed mainly between 68 and $71\,^{\circ }\hbox {C}$, is stabilized at the interface level of VO$_2$ on c-sapphire due to substrate strain effect. The small discrepancy in the temperature range at which the M2 phase is observed in our study compared to that reported in Ref.^34^ could be due to the different conditions of growth that may affect the microstructure of the studied films. In addition, the frequency of the Raman modes at 193 and 613 cm$^{-1}$, considered as a fingerprint for the different VO$_2$ phases, shifts to higher energy when the thickness of the film decreases (see Table 2).

**Table 2:** Observed room-temperature phonon frequencies of undoped and Cr-doped VO$_2$ on c- and r-plane sapphires for thicknesses of 140, 70, 30 and 15 nm^46^.

Raman modes symmetry	VO$_2$ c-plane	Cr-doped VO$_2$ c-plane	VO$_2$ r-plane	Cr-doped VO$_2$ r-plane
	140/70/30/15 nm	140/70/30/15 nm	140/70/30/15 nm	140/70/30/15 nm
†	–/–/–/–	122/126/126/129	–/–/–/–	128/135/137/134
B$_{g}$	140/141/141/137	–/–/–/–	139/139/140/140	–/–/–/–
A$_{g}$	193/194/195/195	199/197/197/197	195/195/196/196	201/197/198/194
A$_{g}$	224/224/224/224	224/224/224/222	224/224/225/225	223/222/224/223
B$_{g}$	262/262/263/260	273/273/272/273	262/262/263/–	272/269/271/–
A$_{g}$/B$_{g}$	308/309/310/309	304/306/305/308	309/308/310/–	304/309/305/301
A$_{g}$/B$_{g}$	338/339/339/341	340/340/340/341	339/335/336/–	341/–/–/–
A$_{g}$	390/391/391/390/	–/–/–/–	390/386/382/–	–/–/–/–
B$_{g}$	–/–/–/–	394/394/–/–/	396/397/399/–	–/–/401/399
^‡^A$_{g}$	435/–/435/–	–/–/–/–	–/–/–/–	438/–/–/–
B$_{g}$	444/443/435/–	446/444/–/–	444/443/–/–	444/–/–/–
B$_{g}$	–/–/–/–	–/–/–/–	–/–/–/–	453/–/–/–
†	–/–/–/–	–/–/–/–	479/477/–/–	–/–/–/–
A$_{g}$	497/496/498/477	501/501/504/–	497/498/491/–	500/495/–/–
A$_{g}$	613/614/615/615	–/–/–/–	616/615/618/619	–/–/–/–
A$_{g}$	–/–/–/–	620/619/–/–	–/–/–/–	–/–/–/–
B$_{g}$	–/–/–/–	646/648/632/630	–/–/–/–	637/626/632/619
^+^A$_{1g}$/B$_{g}$	^+^655/660/662/–	–/–/–/–	664/^+^646/–/–	–/–/–/–
B$_{g}$	820/822/820/–	815/812/816/–	822/824/818/–	823/815/816/815

^†^Raman mode symmetry not yet assigned in literature; ^‡^Raman mode symmetry assigned as A$_g$ by Aronov et al.^56^; ^+^Raman mode symmetry assigned as A$_{1g}$ by Srivastava et al.^42^; and – no peaks found.

In particular, the mode at 193 cm$^{-1}$ approaches the corresponding mode frequencies of M2 phase and the 613 cm$^{-1}$ mode shifts by 2 cm$^{-1}$ while broadening. These observations are in agreement with the findings reported by Atkin et al.^44^ to distinguish the Raman response of the M2 phase from that of the M1 phase. This indicates that the appearance of the intermediate insulator phase (expected to be M2 phase) in the VO$_2$ films grown on c-plane sapphire is due to a structural disorder induced by the in-plane tensile strain along the c$_R$ (i.e., a$_M$) axis that destabilizes the M1 phase. By doping VO$_2$ with ≥ 2.4 at. % Cr, only the M2 phase is present, the large phase separation vanishes and the SPT and MIT temperatures remain similar. For the undoped VO$_2$ on r-plane sapphire substrate in Fig. 7b, no evidence of phase separation is observed and the temperatures corresponding to the structural and electronic transitions are almost the same, attesting the strong coupling between the SPT and the MIT. This connection vanishes (or is reduced) when the film is doped with Cr as seen in Fig. 7d. Interestingly, in this case, the electronic transition precedes the structural transition. The SPT and the MIT in Cr-doped VO$_2$ on r-plane sapphire develop over different temperature scales and are separated by region where an unusual monoclinic-like metallic phase is present. Our finding suggests that the monoclinic-like metallic phase, recently identified in VO$_2$ thin films grown on TiO$_2$(110)^57^, also takes place for Cr-doped VO$_2$ grown on r-plane sapphire. This unusual monoclinic-like metallic phase could be due to a substantial weakening of the V–V bond stabilized by a combination of substrate compressive and doping strain effect, since metal doping of VO$_2$ is known to create an internal strain effect^41^.

**Figure 8:**
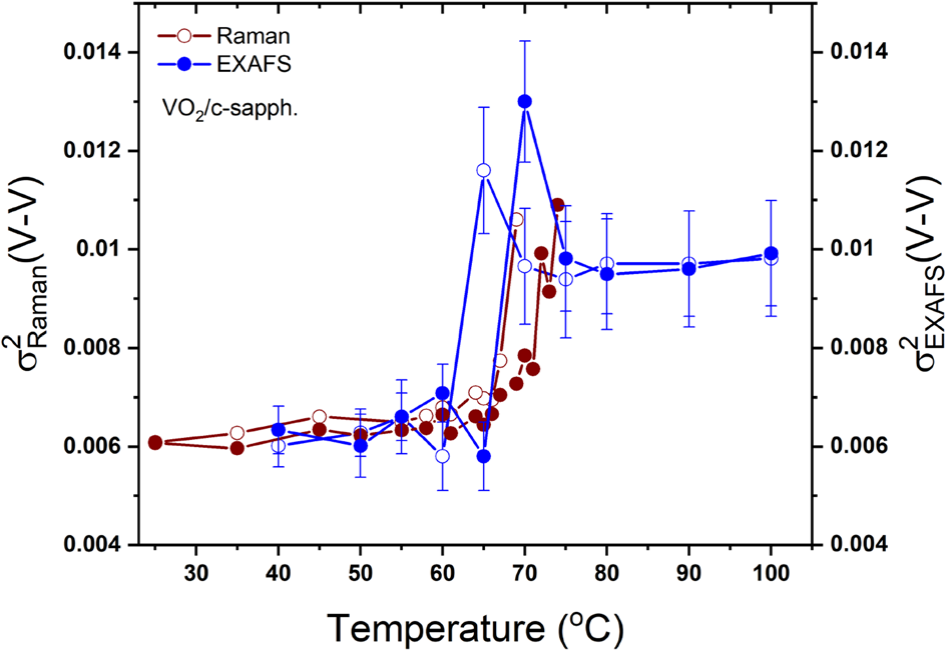
Temperature-dependent mean square relative displacement determined from our Raman data and from EXAFS measurements^21^. Filled symbols correspond to heating cycles and open symbols to cooling cycles. Wine symbols are for Raman measurements and blue symbols for EXAFS.

In order to learn more about the role of the lattice vibration in the metallization of VO$_2$, and the influence of the local structural disorder across the SPT, we combined our Raman data with EXAFS measurements as reported in Ref.^21^. In EXAFS theories, the Debye–Waller describes the temperature dependence of the envelope of the EXAFS oscillations which is expressed in terms of the mean-square relative displacements (MSRD) between the absorbing atom and its near neighbors^58^. The connection between Raman and EXAFS data relies on the fact that the bond-stretching vibration of the atoms, which contributes to the Raman intensity, is essentially the vibrational mechanism determining the EXAFS Debye–Waller factors^25^. The appropriate way to interpret the Raman intensity is therefore to examine the reduced Raman spectrum $I_R(\omega )$, which should be free from temperature dependent factors, compares better with the phonon density of states and is given by

3 $$\begin{aligned} I_R(\omega ) = I(\omega )\omega [n(\omega )+1]^{-1}, \end{aligned}$$

where $n(\omega )$ is the Bose–Einstein distribution factor and $I(\omega )$ is the observed intensity. This reduced Raman intensity can be approximated by the product $I_R (\omega )=\rho (\omega )R(\omega )$, where $\rho (\omega )$ is the density of vibrational states and $R(\omega )$ a matrix element. A best fit for the phonon density of states usually results in $R(\omega )\propto \omega ^2$^23,25,59^. To consider only the contribution of the monoclinic phase of VO$_2$ to the Raman response, we normalized the reduced Raman intensity to the monoclinic phase fraction $m/(m+r) = s_m$^25,58,59^. The nearest neighbor MSRD may be expressed in terms of a relative density of vibrational states (also referred to as local or projected density of vibrational states) to which only the modes giving rise to compression of the bonds contribute. Using the reduced Raman intensity expression, the nearest neighbor MSRD is given by

4 $$\begin{aligned} \sigma _{R}^{2}=\frac{\hslash }{2\mu }\int \frac{I_R(\omega )}{\omega R(\omega )s_m}\coth (\frac{\hslash \omega }{2k_B T})d\omega , \end{aligned}$$

where $\hslash$ is the reduced Planck constant, $\mu$ is the reduced mass of the atomic pair and $k_B$ is the Boltzmann constant. In our calculation of $\sigma _{R}^{2}(V-V)$ of the V–V pair, we considered only the Raman active-modes, at low frequencies, sensitive to the V–V vibrations. The results of our calculation are presented in Fig. 8.

**Figure 9:**
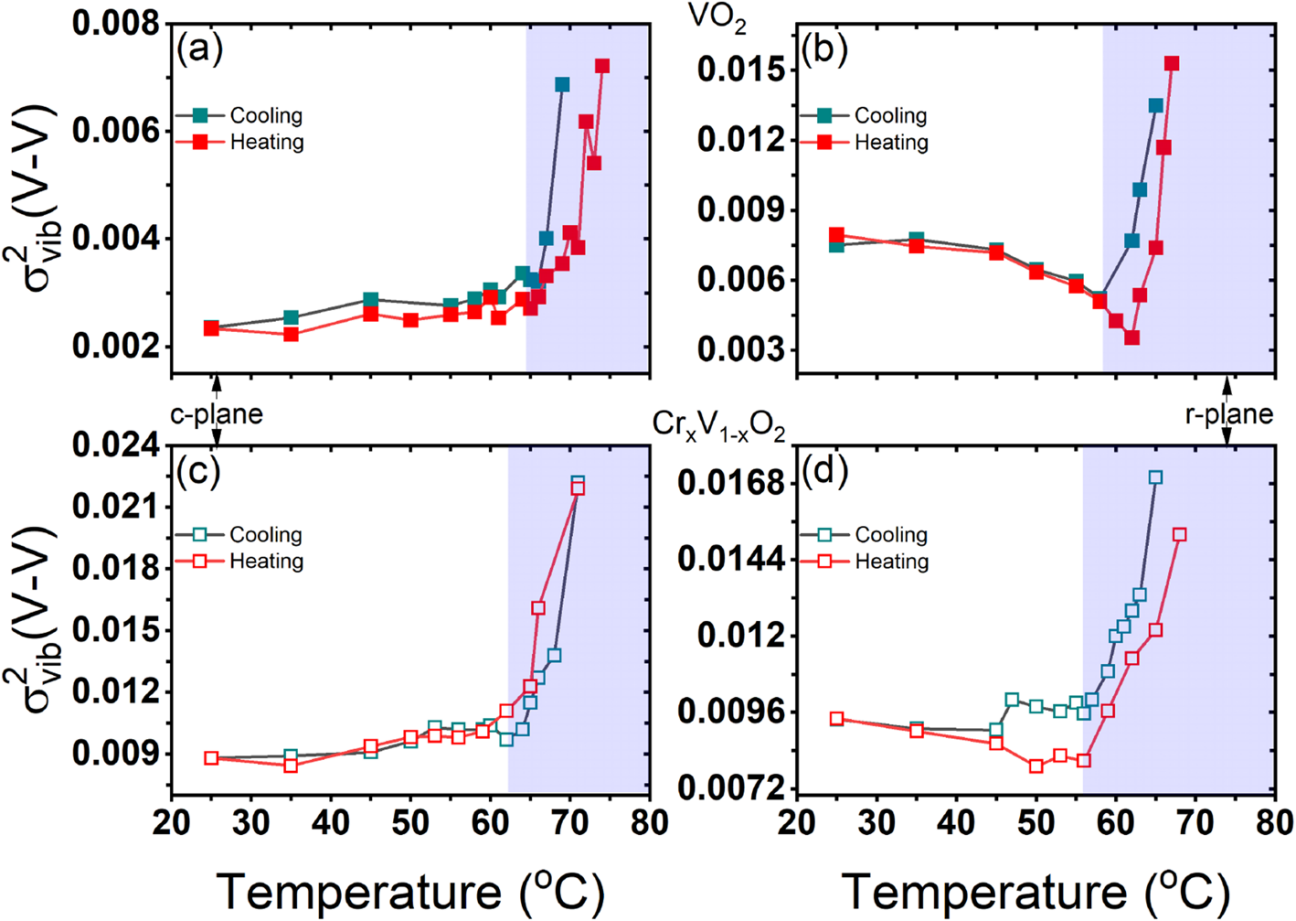
Temperature-dependent $\sigma _{vib}^{2}(V-V)$ determined from our Raman data which is obtained using the same parameters as in Fig. 8. A negligible fraction contribution is not considered.

The temperature dependence of $\sigma _{R}^{2}(V-V)$ obtained from the Raman data is compared with that deduced from the EXAFS measurements of $\sigma _{EXAFS}^{2}(V-V)$ as reported by Hwang et al.^21^ for the VO$_2$ grown on c-plane sapphire. In the fits of the EXAFS data, the disorder parameter $\sigma ^2=\sigma _{sta}^{2}+\sigma _{vib}^{2}$ includes the static disorder $\sigma _{sta}^{2}$ and the thermal vibration disorder $\sigma _{vib}^{2}$. The MSRD $\sigma _{R}^{2}$ obtained from the Raman data represents only the thermal vibration disorder $\sigma _{vib}^{2}$. In Eq. 4, the only adjusting parameter is the proportionality factor between $R(\omega )$ and $\omega ^2$, which is independent of temperature and controls only the $\sigma _{R}^{2}$ magnitude. By adjusting this factor to obtain the same $\Delta \sigma _{R}^{2}(V-V)$ shift, we can estimate the static disorder, $\sigma _{sta}^{2}$, which is $\sim$ 0.004 Å$^{2}$ ($\sigma _{sta} \sim$ 0.063 Å). The temperature dependence of $\sigma _{R}^{2}(V-V)$, obtained from the Raman data, is in agreement with that of $\sigma _{EXAFS}^{2}(V-V)$ deduced from the EXAFS analysis, exhibiting a slight shift of the transition temperature, which is probably due to the intrinsic property of each studied film (thickness, microstructure and growth conditions). Interestingly, both $\sigma _{R}^{2}(V-V)$ and $\sigma _{EXAFS}^{2}(V-V)$ show an unexpected increase around the transition temperature. Indeed, previous theoretical and experimental studies have shown that the rutile phase is structurally more stable than the monoclinic phase^60^. Hence, it is expected that the disorder parameter $\sigma ^2$ of the V–V pairs should be larger in the monoclinic phase than in the rutile phase, since the zigzag configuration of the V atoms in the M phases can induce more static disorder in the atomic V–V pairs. Moreover, an additional structural disorder is expected in the monoclinic phase since the VO$_2$ films, initially grown at $550\,^{\circ }\hbox {C}$, are stabilized in the rutile phase and cooled down to the monoclinic phase at room temperature. While EXAFS measurements of $\sigma ^2$ cannot separate between the static disorder and the vibrational disorder contributions but, here, by calculating the MSRD $\sigma _{R}^{2}$ from vibrational Raman data, we clearly reveal (see Fig. 8) that the unexpected increase of $\sigma ^2$ is not due to a static disorder but is rather related to the thermal vibration contribution, which gradually increases (decreases) during heating (cooling). Our findings provide an evidence on the role of the thermal vibrational disorder in the SPT in VO$_2$ and, accordingly, in its MIT. In particular, our results indicate that the presence of an intermediate insulator structure, the M2 phase for VO$_2$ grown on c-plane sapphire, is mainly related to a vibrational structural disorder, particularly at the V sites. This structural disorder could be sensitive to the in-plane tensile strain induced on the film by the c-plane sapphire. To calculate the thermal vibration disorder, $\sigma _{vib}^{2}(V-V)$ in VO$_2$ and Cr-doped VO$_2$ on c- and r-plane sapphire substrates, we have extrapolated the same analysis on the Raman data from Fig. 6 and the results of our calculation are presented in Fig. 9. We found that, generally, $\sigma _{vib}^{2}(V-V)$ increases across the SPT in all the studied films attesting to the key role of the thermal vibration disorder in VO$_2$ phase transition. Further investigations are currently underway on VO$_2$ polycrystalline films to understand the effect of oxygen pressure during deposition and VO$_2$ films Cr content on this thermal vibrational disorder.

In summary, we have performed a systematic Raman study of the phase transitions in undoped and Cr-doped VO$_2$ thin films. The coupling/decoupling between the SPT and the MIT in the studied films was analyzed by quantitatively comparing the structural fraction analyses, calculated from Raman data, to the metallic fraction obtained from electrical measurements. Our results show that the coupling between the structural and the electronic transitions in VO$_2$ films is sensitive to the substrate strain (tensile or compressive strain) and also to the involved transition (M1-R or M2-R). Interestingly, our findings suggest that the monoclinic-like metallic phase, recently identified at ambient pressure in VO$_2$ thin films grown on TiO$_2$ (110), is also taking place in the Cr-doped VO$_2$ grown on r-plane sapphire. We have also established a connection between the Raman intensities of the VO$_2$ films and the mean-square relative displacements $\sigma ^2$, that determines the Debye–Waller factor in the EXAFS analysis and found that the temperature dependence of the $\sigma _{R}^{2}(V-V)$ as obtained from the Raman data, reproduces the temperature profile of the $\sigma _{EXAFS}^{2}(V-V)$ deduced from the EXAFS data. Our analysis demonstrates that the thermal vibrational disorder, not considered in previous theoretical works, play an important role in the phase transition of VO$_2$ films. This effect, expected to exist in a wide variety of strongly correlated electron materials, such as IrTe$_2$^61^, can be explored with this combination approach between Raman and EXAFS data.

## Methods

Undoped and Cr-doped VO$_2$ thin films were deposited on r- and c-sapphire substrates using reactive pulsed laser deposition (RPLD). The undoped and 5% Cr-doped vanadium targets were mounted in a vacuum chamber (base pressure of $10^{-6}$ Torr) at a distance of 6.5 cm from the substrate and ablated using a KrF excimer laser ($\lambda$ = 248 nm, fluence of  2 J/cm$^2$, repetition rate of 10 Hz). Both the substrates and the targets were rotated and the laser beam was rastered over the target surface by the constant translation of a focusing lens to achieve good film homogeneity. The temperature was maintained at $550\,^{\circ }\hbox {C}$ and the oxygen pressure at 22 mTorr during the deposition process. Consistent thickness of the films was achieved through a proper optimization of the deposition rate. The structural properties were examined by x-ray diffraction (XRD) in the $\theta$-2$\theta$ configuration of a PANalytical X’Pert PRO diffractometer with Cu K$\alpha$ radiation operated at 45 kV and 40 mA. The morphological and electrical characterizations were performed respectively using a JEOL JSM-6300F scanning electron microscopy (SEM) and a standard four point-probe measurement^62^. The stoichiometry of the films was Cr$_x$V$_{1-x}$O$_2$ (*x* = 3) as determined using Rutherford backscattering spectrometry (RBS) measurements. Temperature-dependent unpolarized Raman spectroscopy measurements were performed with a Renishaw inVia Reflex confocal equipped with a $\lambda$ = 514 nm excitation laser, a 1800 lines/mm grating and a 1 cm$^{-1}$ spectral resolution. The laser power was about 500 $\upmu$W to avoid any local heating of the samples and possible modification of their structure. The temperature was controlled by Linkam 600 thermal stage with a silver heating/cooling element, providing temperature stability of ± $0.1\,^{\circ }\hbox {C}$. A long working distance objective lens of $\times$50 magnification with a numerical aperture of 0.5 was used for the measurements.

## Supplementary Information

Supplementary Information.
