# Supplementary material for: Probing the role of thermal vibrational disorder in the SPT of VO2 by Raman spectroscopy
Source: Sci Rep. 2021 Jan 15;11:1620. doi: 10.1038/s41598-020-79758-1 (PMC7810880; doi:10.1038/s41598-020-79758-1)
Supplement: Supplementary file 1 — Supplementary Information. [file 41598_2020_79758_MOESM1_ESM.pdf]

Probing the role of thermal vibrational disorder in the SPT of VO_2 by Raman spectroscopy

Aminat Oyiza Suleiman[1,+,*], Sabeur Mansouri[1,+,*], Nicolas Émond[2], Boris Le Drogoff[1], Théophile Bégin[3], Joëlle Margot[3], and Mohamed Chaker[1,*]

[1]Institut National de la Recherche Scientifique, Énergie Matériaux Télécommunications, 1650, Boulevard Lionel-Boulet, Varennes, QC, J3X 1S2 Canada
[2]Department of Materials Science and Engineering, Massachusetts Institute of Technology, 77 Massachusetts Avenue, Cambridge, MA, 02139, USA
[3]Département de Physique, Complexe des Sciences, Université de Montréal, 1375 Avenue Thérèse-Lavoie-Roux, Montréal, QC H2V 0B3, Canada
[*]Corresponding. chaker@emt.inrs.ca
[*]Corresponding. aminat.suleiman@emt.inrs.ca
[*]Corresponding. sabeur.mansouri@emt.inrs.ca
[+]These authors contributed equally to this work.

1 Supplementary Material

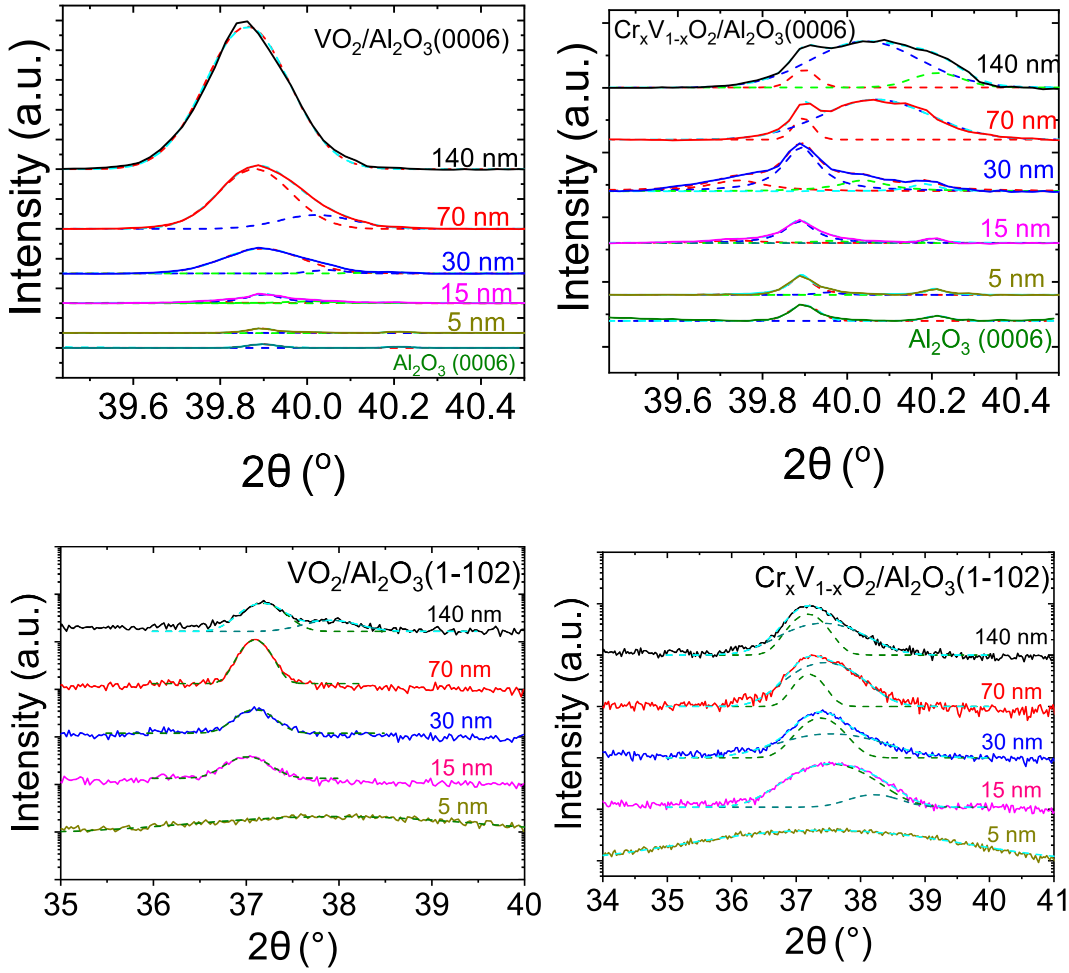

Figure S1-1. Thickness dependent XRD peak spectra of undoped and Cr-doped VO_2 on (a) c-plane and (b) r-plane sapphire substrates.

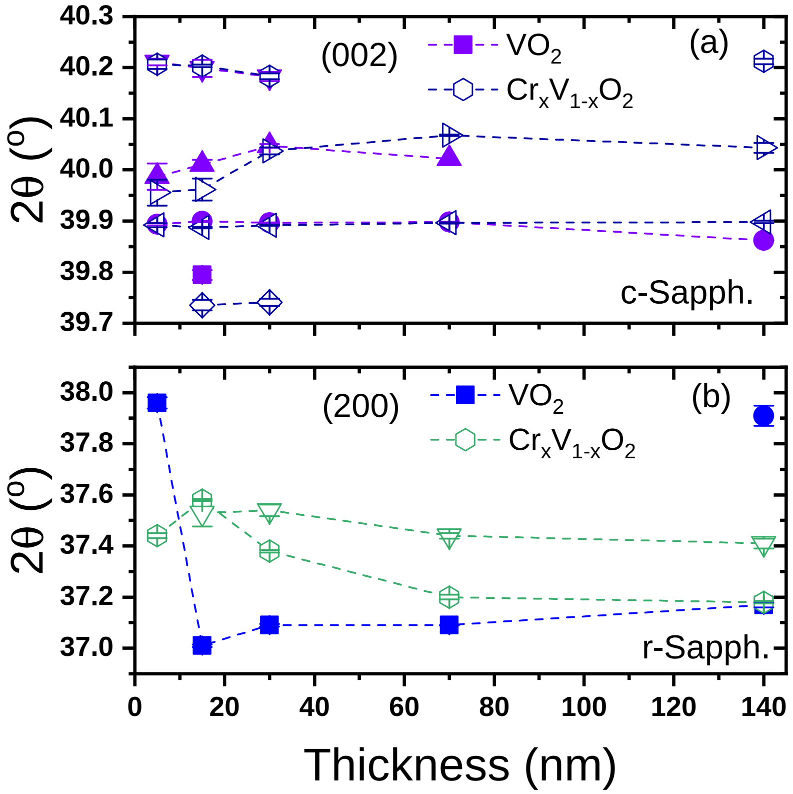

Figure S1-2. Thickness dependent peak positions of undoped and Cr-doped VO_2 on (a) c-plane and (b) r-plane sapphire substrates.

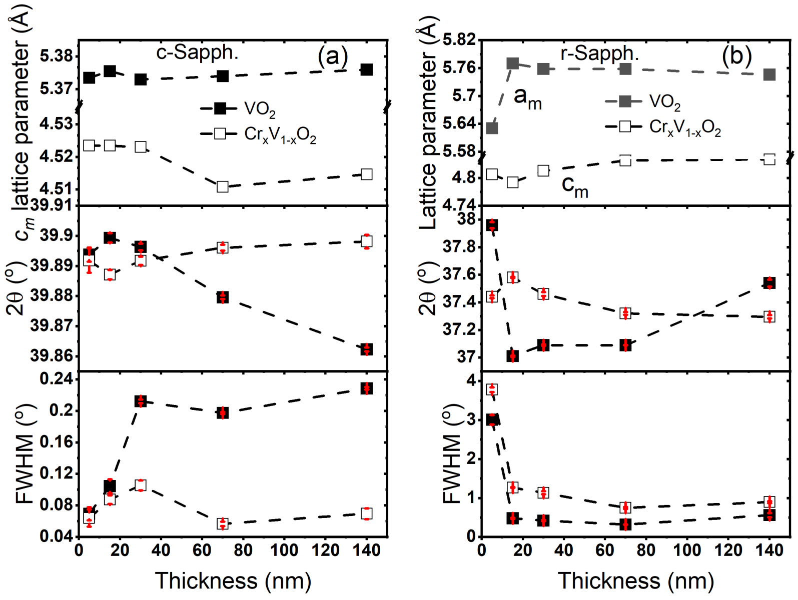

Figure S1-3. The peak positions, full-width-half-maximum (FWHM) and lattice parameters undoped and Cr-doped VO_2 on (a) c-plane and (b) r-plane sapphire substrates.

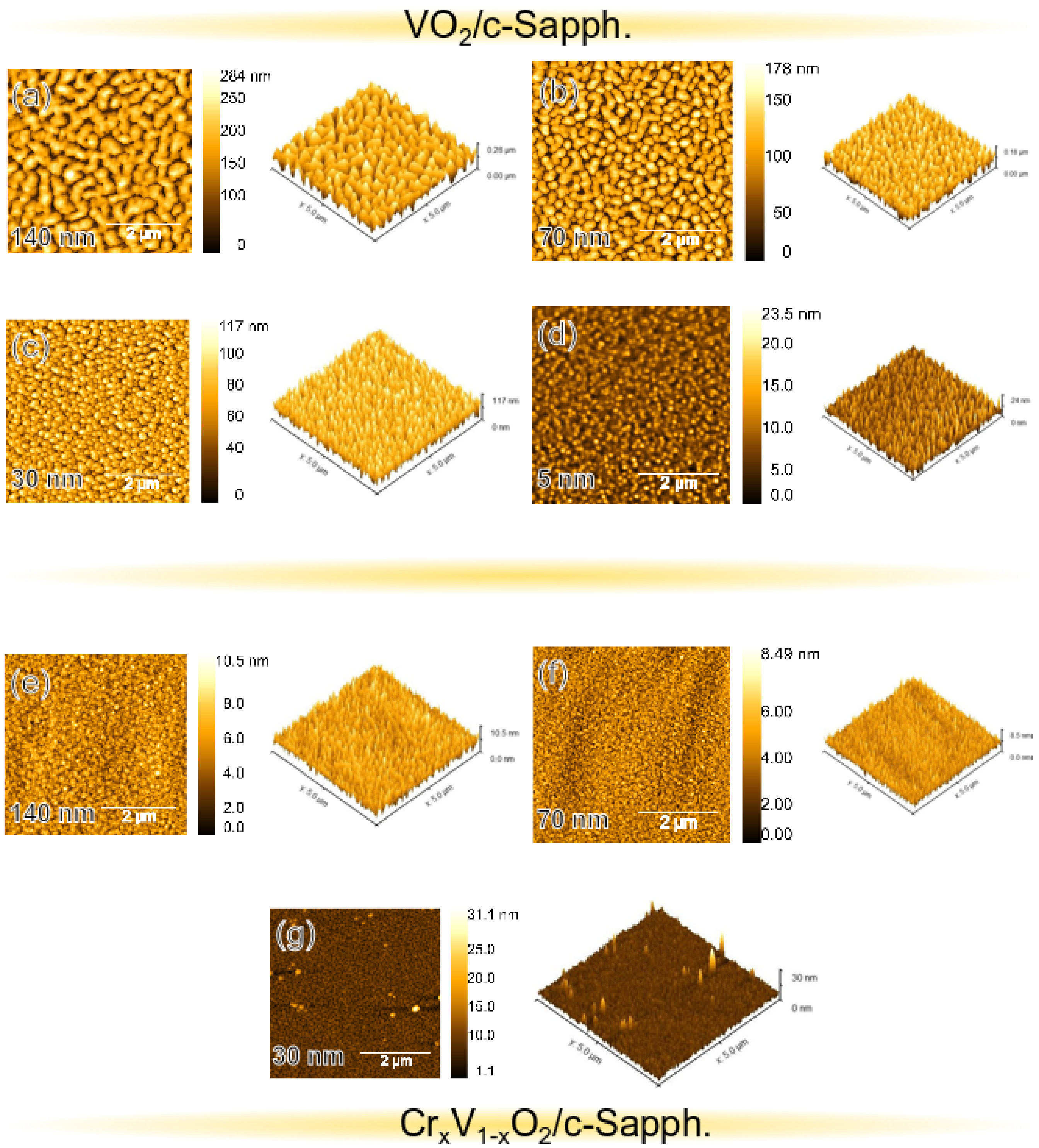

Figure S1-4. AFM surface morphology of undoped (a)-(d) and Cr-doped VO_2 (e)-(g) on c-plane sapphire substrate.

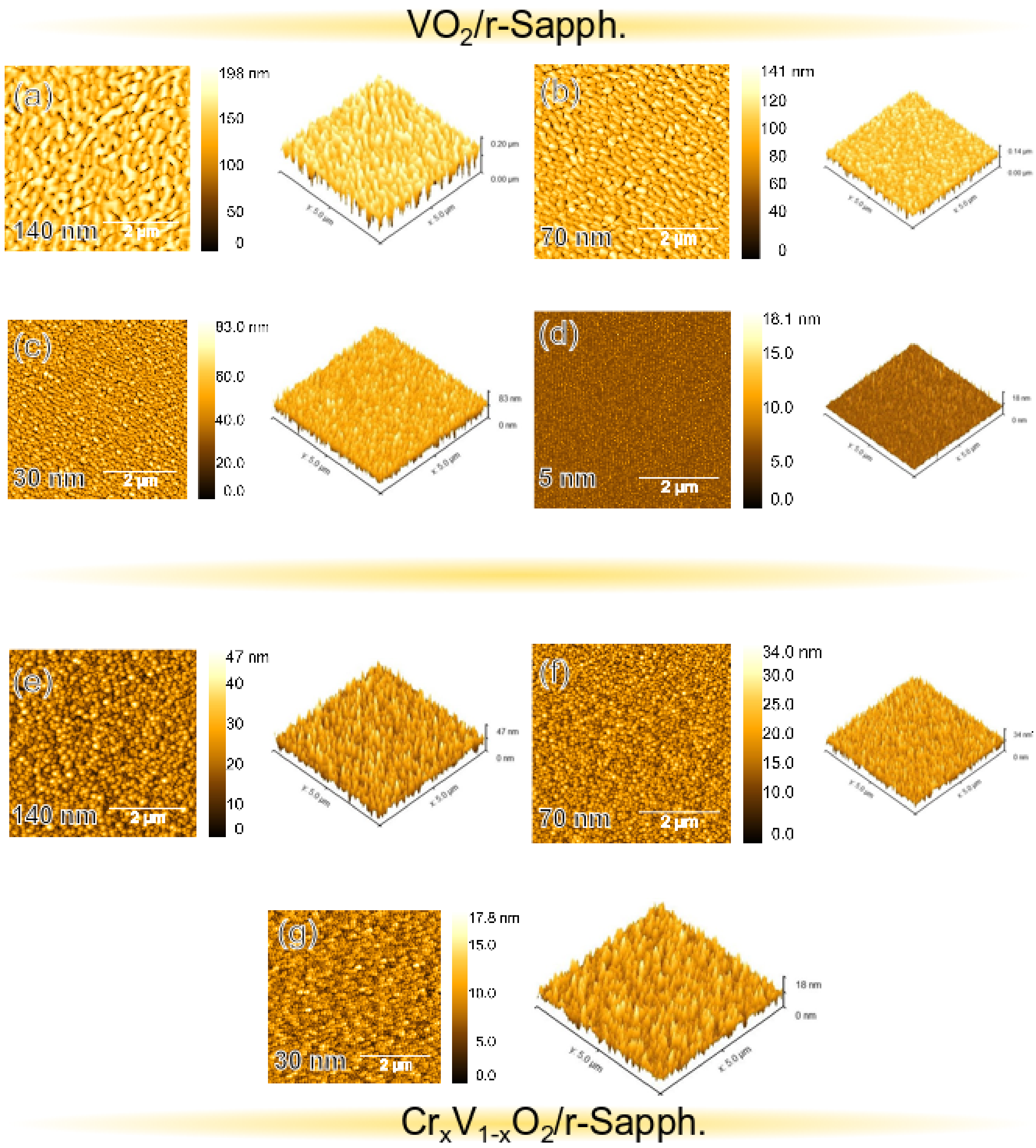

Figure S1-5. AFM surface morphology of undoped (a)-(d) and Cr-doped VO_2 (e)-(g) on r-plane sapphire substrate.

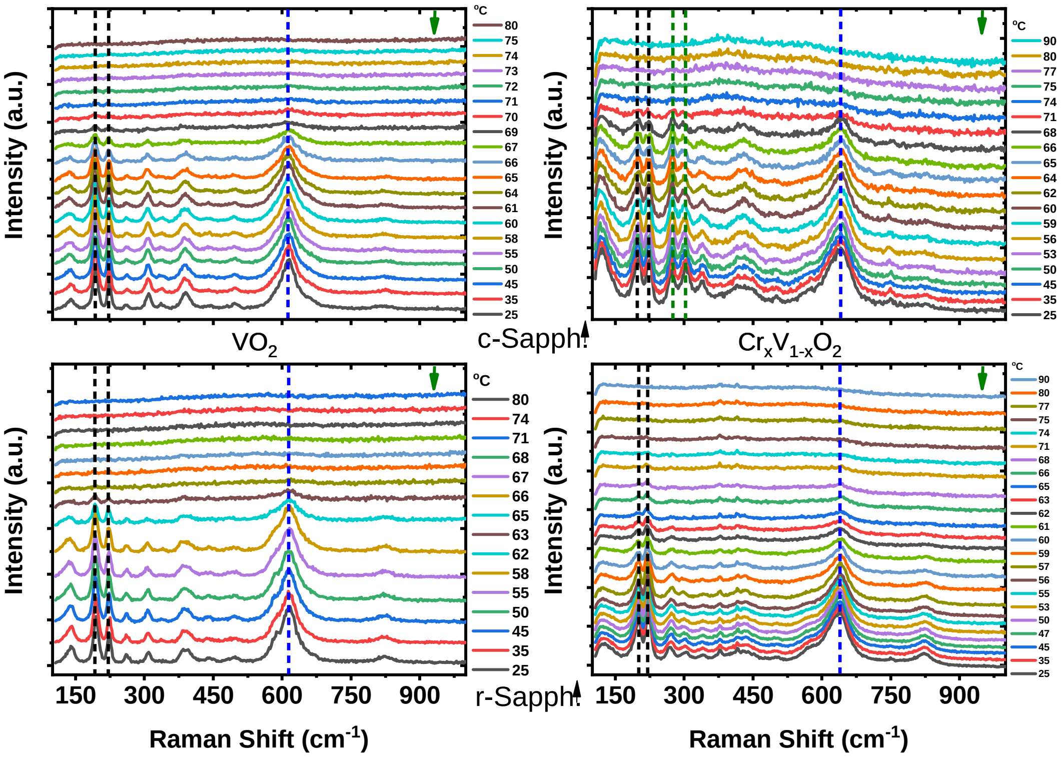

Figure S1-6. The Raman spectra of undoped and Cr-doped VO_2 on c-plane and r-plane sapphire substrates with the green arrow indicating the thermal cycle of cooling. The dotted black and blue lines depict the Raman peak positions of the low (V-V) and the high (V-O) frequency Raman modes respectively.
